# Efficacy and safety of acetyl-CoA carboxylase (ACC) inhibitors in the treatment of nonalcoholic steatohepatitis (NASH): A protocol for systematic review

**DOI:** 10.1097/MD.0000000000032357

**Published:** 2022-12-16

**Authors:** Liubin Xu, Huili Yang, Hongling Xu, Rong Yang, Lian Fen, Dansheng Jiang, Linyi Xu, Yufeng Xing

**Affiliations:** a Department of Hepatology, Shenzhen Traditional Chinese Medicine Hospital, Shenzhen, China; b The Fourth Clinical Medical College of Guangzhou University of Chinese Medicine, Shenzhen, China.

**Keywords:** acetyl-CoA carboxylase inhibitors, nonalcoholic steatohepatitis, protocol, systematic review

## Abstract

The pathological mechanism of nonalcoholic steatohepatitis (NASH) is closely related to abnormal lipid regulation in hepatocytes. Patients with NASH generally have a significant increase in *de novo lipogenesis*, which acetyl-CoA carboxylase (ACC) catalyzes the first committed step. However, the treatment with ACC inhibitors remains controversial. Thus, our study will systematically evaluate the efficacy and safety of ACC inhibitors for the treatment of NASH.

**Methods:**

We plan to search PubMed, Cochrane Library, Web of Science, EMBASE, Google Scholar, ClinicalTrials.gov, China Science and Technology Journal Database, Chinese Biomedical Literature Database, Wan-fang Database and China National Knowledge Infrastructure to obtain literatures from January 2015 to January 2030 under the inclusion and exclusion criteria, and include randomized controlled trials containing intervention of ACC inhibitors for NASH. The proportion of patients with reduction in ballooning, inflammation and fibrosis will be accepted as the main outcome. RoB 2 will be used for the risk of bias, as well as Egger’s test and funnel plot for reporting bias. We will adopt Review Manager 5.4.1 for data synthesis, subgroup analysis, meta-regression analysis and sensitivity analysis, and conduct trial sequential analysis and quality of evidence evaluation using trial sequential analysis 0.9.5.10 Beta software and GRADE Profiler 3.6.1 software respectively.

**Results:**

This systematic review will assess the proportion of patients with reduction of ballooning, inflammation and fibrosis, changes in hepatic steatosis, levels of liver enzymes and liver injury markers, metabolic parameters, safety and tolerability to measure the clinical benefits of ACC inhibitors for NASH.

**Conclusion:**

The conclusion of this systematic review will achieve convincing evidence to evaluate the efficacy and safety of ACC inhibitors for NASH.

## 1. Introduction

Nonalcoholic fatty liver disease (NAFLD) affects more than 25% of people worldwide and is the most common chronic liver disease by far.^[1]^ Among them, 10% to 30% of patients with NAFLD can be diagnosed as nonalcoholic steatohepatitis (NASH), of which pathological features include hepatic steatosis, inflammation, balloonlike degeneration and fibrosis.^[2,3]^ As a vital part of the natural history of NAFLD, NASH has a tendency to develop into liver cirrhosis and even hepatocellular carcinoma, and has become the main cause of end-stage liver disease needing liver transplantation.^[4]^ According to statistics, among NASH patients with fibrosis, the 3-year incidence of HCC was 12.8%.^[5]^ Although the medical burden caused by NASH is great, there is still no drug approved by Food and Drug Administration for the treatment of NASH.^[6]^

*De novo lipogenesis* (DNL), which promotes the accumulation of triglycerides (TG) in hepatocytes and causes persistent steatosis leading to lipid toxicity, inflammation, and fibrosis, is significantly increased in NASH patients.^[7]^ Acetyl-CoA carboxylase (ACC), a rate-limiting enzyme in DNL, has ACC1 and ACC2 subtypes which are key enzymes in fatty acid (FA) synthesis and oxidation respectively.^[8]^ ACC can catalyze the formation of acetyl-CoA into malonyl-CoA, which is the main substrate for FA biosynthesis as well as an allosteric inhibitor of carnitine palmityl transferase, and carnitine palmityl transferase can mediate FAs into mitochondria for *β*-oxidation.^[9,10]^ Therefore, ACC inhibitors are expected to alleviate NASH by reducing DNL and enhancing mitochondrial *β*-oxidation. A randomized controlled trial (RCT) conducted by Stiede et al confirmed that GS-0976, an ACC inhibitor, at 20 mg, 50 mg and 200 mg doses significantly inhibited DNL by 70%, 85% and 104% respectively.^[11]^ Another 12-week clinical trial showed that the oral administration of 20 mg GS-0976 daily had a significant improvement in liver fat content and stiffness, with a ≥ 30% decrease in magnetic resonance imaging proton density fat fraction (MRI-PDFF) among 70% of patients and a 9% reduction on magnetic resonance elastography, while 2 patients had abnormal elevated alanine aminotransferase (ALT) and TG, respectively.^[12]^ Similarly, in a Phase 2 NASH trial, 20 mg GS-0976 was shown to significantly reduce hepatic steatosis (as measured by MRI-PDFF) by ≥ 30% in nearly half of patients, but more patients developed hypertriglyceridemia during treatment.^[13]^ The above studies suggest that ACC inhibitors have significant efficacy in improving liver steatosis in NASH patients, but its safety remains controversial.

Up to now, there is still a lack of systematic evaluation to definitively confirm the clinical efficacy of ACC inhibitors for NASH. Therefore, our study aims to systematically illustrate the effectiveness and safety of ACC inhibitors in the treatment of NASH by summarizing the published RCTs in Both Chinese and English databases, and to provide theoretical basis or guidance for future research and clinical treatment.

## 2. Methods

### 2.1. Registration

The protocol of our research has been registered in the international prospective register of systematic reviews (PROSPERO) and given a PROSPERO registration number of CRD 42020167735.

### 2.2. Ethics and dissemination

As the date needed will come from published articles instead of patients, there is no requirement for ethical approval in this research. This system assessment achievements will be published in a peer-reviewed journal with the clarification of the effectiveness and safety of ACC inhibitors in the treatment of NASH, and thus provide reference for clinicians to make treatment decisions.

### 2.3. Inclusion criteria

#### 2.3.1. Participants.

*Eligibility criteria*: age 18 to 75 years; overweight or obese with a body mass index > 25kg/m^2^; liver biopsy conducted within 3 months after screening showed histological evidence of steatohepatitis with a NAFLD activity score ≥ 4 (at least 1 subscore in each subcomponent of hepatocyte ballooning, steatosis and lobular inflammation and) and a fibrosis stage of 1, 2, or 3;^[14]^ MRI-PDFF ≥ 8%; elevated ALT (> 30 IU/L for men or 19 IU/L for women).

*Ineligibility criteria*: hepatitis caused by virus, alcohol, autoimmune liver disease and other factors; liver cirrhosis; liver transplantation history; pregnant or lactating women; malignant tumors, cardiovascular diseases and other related major diseases; international normalized ratio > 1.2; conjugated bilirubin ≥ 35µmol/L; serum albumin ≤ 3.2g/ dL; ALT > 5 × the upper limit of normal; serum creatinine ≤ 2 mg/dL.

#### 2.3.2. Interventions.

Patients in the experimental group receive ACC inhibitors (GS-0976/NDI-010976/ND-630/PF-05221304/Firsocostat) orally once daily, while in the control group received placebo.

#### 2.3.3. Endpoint.

The primary endpoint is the proportion of patients with reduction of ballooning, inflammation and fibrosis assessed by liver biopsy or noninvasive tests (transient elastography and magnetic resonance elastography).^[15,16]^ Secondary endpoints are changes in hepatic steatosis measured by MRI-PDFF and controlled attenuation parameter, levels of liver enzymes and liver injury markers, metabolic parameters (weight, blood glucose and lipid levels), safety and tolerability (incidence of adverse events, relevant laboratory tests, vital sign measurements).

#### 2.3.4. Study design.

RCTs containing intervention groups of ACC inhibitors for NASH and control groups will be integrated into this study, regardless of sample size, publication status, or language.

### 2.4. Exclusion criteria

Non-randomized clinical controlled trials, for instance, reviews, conference articles, case reports, observational researches and repeated reports will not be taken into account.

### 2.5. Search strategy

RCTs, of which published time ranging from January 2015 to January 2030, involved in this study will be obtained by searching PubMed, Cochrane Library, Web of Science, EMBASE, Google Scholar, ClinicalTrials.gov, China Science and Technology Journal Database, Chinese Biomedical Literature Database, Wan-fang Database and China National Knowledge Infrastructure under the inclusion criteria. The search block of *P* + *I* + *S* will be retrieved by combining with the medical subject headings search and text word search. Search words will be connected with “OR” to form search items and then logically assembled with “AND.” We will also adjust the title, keyword and summary fields based on the features of different databases. The search terms for PubMed are shown in Table 1.

**Table 1:** Search items.

Search block	Key search terms
Participants	“Nonalcoholic steatohepatitis [MeSH Terms]” OR “Nonalcoholic steatohepatitis* [Title/Abstract]” OR “NASH [Title/Abstract]”
Interventions	“Acetyl-CoA carboxylase inhibitor [MeSH Terms]” OR “Acetyl-CoA carboxylase inhibitor [Title/Abstract]” OR “ACC inhibitor [Title/Abstract]” OR “GS-0976 [Title/Abstract]” OR “NDI-010976 [Title/Abstract]” OR “ND-630 [Title/Abstract]” OR “PF-05221304 [Title/Abstract]” OR “Firsocostat [Title/Abstract]”
Study design	“Randomized controlled trial [pt]” OR “Controlled clinical trial [pt]” OR “Clinical trial as topic [MeSH: noexp]” OR “Randomized [Title/Abstract]” OR “Placebo [Title/Abstract]” OR “Randomly [Title/Abstract]” OR “Trail [Title/Abstract]”

ACC = acetyl-CoA carboxylase, MeSH = medical subject headings, NASH = nonalcoholic steatohepatitis.

### 2.6. Study selection and data extraction

#### 2.6.1. Study selection.

**Figure 1.**
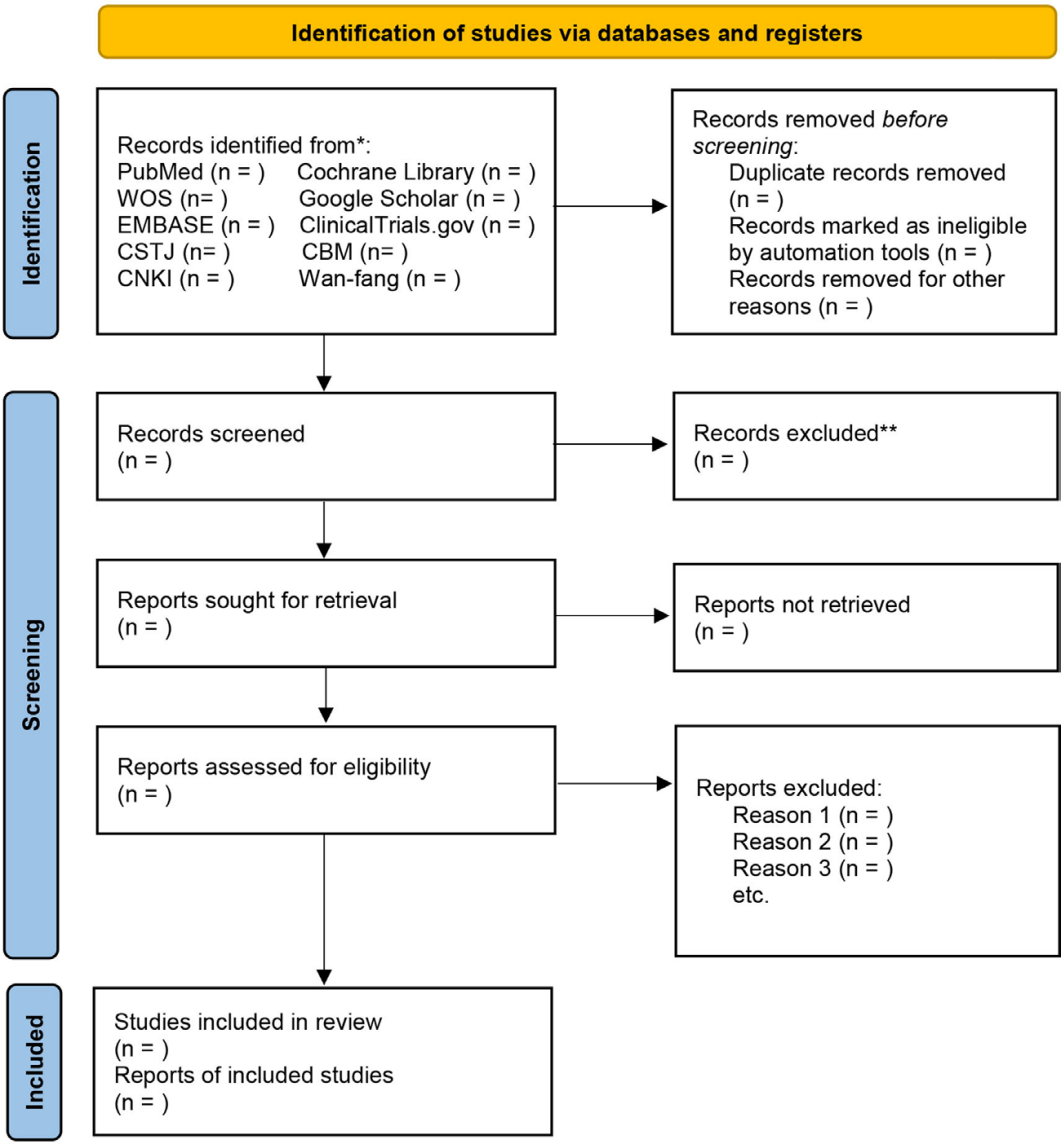
PRISMA flow diagram of study selection process in the systematic review.

Literatures will be screened preliminary according to the inclusion criteria by 2 researchers who will first eliminate duplicate literatures, and then remove clearly ineligible literatures through scanning titles and abstracts. Next, a third researcher will review the full text of the literatures that the 2 researchers had different opinions on whether to exclude in the previous step, and organize a group discussion to resolve divergences, during which, reasons for excluding papers will be recorded. The preferred reporting items for systematic review and meta-analysis (PRISMA) flow diagram exhibits our process of filtrating eligible literatures (Fig.1).

#### 2.6.2. Data extraction.

This study will create a standardized datasheet following the common items in the Cochrane Handbook for Systematic Reviews of Interventions to summarize the important information from the included papers.^[17]^ The data extraction will be first performed independently by 2 researchers, and then a third researcher will act as an arbitrator to assess all data and disagreements arising from the process. The extracted contents include the title, journal, date of publication, first author, research design and duration, sample size, age, sex, disease situation, intervention measures, specifics of outcome indexes and adverse events. In case of data incompleteness, researchers need to contact the primary author to obtain the missing data by means of e-mail or phone. In the event that missing data remain unavailable, we will analyze the possible impact of the missing data on this study.

### 2.7. Risk of bias assessment

Selective bias (stochastic sequence generation and allocation concealment), performance bias (blinding of participants and researchers), measurement bias (blinding of outcome evaluator), follow-up bias (incomplete outcome data), reporting bias (selective reporting), and other biases are the main sources of risk of bias. Two researchers will respectively use RoB 2, a revised tool for assessing risk of bias in randomized trials, to evaluate the above items and grade them to be “high risk,” “low risk,” or “indefinite risk.”^[18]^ In addition, Egger’s test and funnel plot will be used to assess reporting bias when more than 10 articles are included. Supposing that there are differences on the classification of risk level, the final risk level will be determined by discussing or a third evaluator in case of no results from the discussion.

### 2.8. Data synthesis

Review Manager 5.4.1 will be adopted for data synthesis. Tests for heterogeneity between researches will be conducted through the *I*-squared (*I*^*2*^) statistic.^[19]^ *I*^*2*^ ≤ 50% implies acceptable heterogeneity and we will use the fixed-effects model to process statistics data, otherwise the random effects model. *Z* test or *Chi* square test will be used to examine the effect magnitude which is regarded as no statistically significant when *P* > .05. Mean differences (MD) or risk ratios with 95% confidence intervals will be applied to present the results.

### 2.9. Subgroup analysis, meta-regression analysis and sensitivity analysis

If *I*^*2*^ indicates significant heterogeneity, subgroup analysis and meta-regression will be used to explore the source of heterogeneity.^[20]^ Subgroup analysis will be conducted by dividing included researches into different subgroups according to the causes of heterogeneity such as study protocol, intervention measures, control selection, age, sex, duration and disease severity to evaluate the effect value respectively, while meta-regression analysis adding the above factors into the regression model to explain heterogeneity. Sensitivity analysis will be used to evaluate the reliability of meta-analysis results. By comparing the results of meta-analysis which is redone after changing the inclusion criteria, adopting different statistical models, and excluding low-quality research or stratified meta-analysis based on the sample size with previous results, the stability of the results and the impact of excluded researches on effect size will be discussed.

### 2.10. Trial Sequential Analysis (TSA)

TSA 0.9.5.10 Beta software will be used to conduct TSA on the overall effective rate to evaluate whether the evidence is reliable and test whether the cumulative sample size reaches the required information size with statistical indices of *α* = 0.05 for type I error and *β* = 0.20 for type II errors.^[21]^

### 2.11. Quality of evidence

The Grading of Recommendations Assessment, Development, and Evaluation (GRADE) will be used to grade the evidence level into high, medium, low and extremely low through GRADE Profiler 3.6.1 software.^[22]^ We will adjust the level of evidence based on factors including limitations in study design and implementation, circumstantial evidence, reporting bias, unaccountable heterogeneity or inconsistent results, and inaccurate results due to sample size.

## 3. Discussion

NASH is a disease with complex pathophysiology and mechanisms involving multifactorial pathways such as metabolic abnormalities, lipotoxicity, inflammation and fibrogenesis, resulting in very limited pharmacological treatment options for NASH.^[23]^ With the increasing understanding of the mechanisms that lead to the development and progression of NASH, many medical therapies for NASH are being developed around targets of inflammation or cell injury, metabolism, liver–gut axis and anti-fibrosis.^[24]^ As lipotoxicity has been considered to be the key driving factor for the development of NASH and fibrosis, improving liver fibrosis may require drugs that target upstream lipotoxicity and inflammation, and thus ACC is an attractive metabolic target to inhibit DNL and improve mitochondrial fatty acid oxidation.^[7]^ In fact, ACC inhibitors have shown good effects on DNL, liver steatosis, fibrosis markers and liver biochemical indicators in clinical trials.^[11,24,25]^ However, its safety still needs further investigation. Studies have shown that using ACC inhibitors might cause clinically asymptomatic hypertriglyceridemia, headache, nausea, abdominal pain and diarrhea, while the increase of serum TG was thought to result from the adaptive up-regulation of steroid regulatory element binding protein-1, the adipogenic transcription factor in the liver.^[13,26]^ We believe that if there are more high-quality clinical evidences to demonstrate the benefits of ACC inhibitors, the development of treatment options on NASH will be promoted. To date, however, there has been no systematic evaluation of the clinical evidence on the treatment of NASH with ACC inhibitors. Consequently, we plan to evaluate the efficacy and safety of ACC inhibitors through this study to clarify its clinical benefits for NASH. Even so, limitations such as heterogeneity and reporting bias are unavoidable problems in this systematic review, which require us to gain and analyze more studies and results.

## Author contributions

Liubin Xu, Huili Yang and Yufeng Xing designed the study. Liubin Xu, Huili Yang and Hongling Xu drafted the protocol. All authors revised this manuscript and confirmed the final version.

**Conceptualization:** Liubin Xu, Huili Yang, Yufeng Xing.

**Data curation:** Liubin Xu, Huili Yang, Hongling Xu, Rong Yang, Yufeng Xing.

**Formal analysis:** Liubin Xu, Huili Yang.

**Investigation:** Hongling Xu, Rong Yang, Lian Fen.

**Methodology:** Liubin Xu, Huili Yang, Hongling Xu, Yufeng Xing.

**Project administration:** Liubin Xu, Yufeng Xing.

**Resources:** Yufeng Xing.

**Software:** Liubin Xu.

**Supervision:** Yufeng Xing.

**Validation:** Yufeng Xing.

**Visualization:** Liubin Xu, Huili Yang, Dansheng Jiang and Linyi Xu.

**Writing – original draft:** Liubin Xu, Huili Yang.

**Writing – review and editing:** Liubin Xu, Yufeng Xing.
